# New Open-Source Tools: Using Bonsai for Behavioral Tracking and Closed-Loop Experiments

**DOI:** 10.3389/fnbeh.2021.647640

**Published:** 2021-03-31

**Authors:** Gonçalo Lopes, Patricia Monteiro

**Affiliations:** ^1^NeuroGEARS Limited, London, United Kingdom; ^2^Life and Health Sciences Research Institute (ICVS), School of Medicine, University of Minho, Braga, Portugal; ^3^ICVS/3B’s–PT Government Associate Laboratory, Braga/Guimaraes, Portugal

**Keywords:** behavior, neuroscience, open source, visual programming, software

## Abstract

The ability to dynamically control a behavioral task based on real-time animal behavior is an important feature for experimental neuroscientists. However, designing automated boxes for behavioral studies requires a coordinated combination of mechanical, electronic, and software design skills which can challenge even the best engineers, and for that reason used to be out of reach for the majority of experimental neurobiology and behavioral pharmacology researchers. Due to parallel advances in open-source hardware and software developed for neuroscience researchers, by neuroscience researchers, the landscape has now changed significantly. Here, we discuss powerful approaches to the study of behavior using examples and tutorials in the Bonsai visual programming language, towards designing simple neuroscience experiments that can help researchers immediately get started. This language makes it easy for researchers, even without programming experience, to combine the operation of several open-source devices in parallel and design their own integrated custom solutions, enabling unique and flexible approaches to the study of behavior, including video tracking of behavior and closed-loop electrophysiology.

## Introduction

Quantifying animal behavior is crucial in many fields of biological research such as behavioral pharmacology, neuroscience, or ecology. By observing animal behavior in diverse settings, researchers try to extract information about internal states, aiming to understand the causal structure and dynamic properties of genetic and environmental factors (Gomez-Marin et al., 2014; Krakauer et al., 2017).

It is common to consider behavior as simply the set of all movements exhibited by an animal over time, and that the goal of the researcher is having the ability to predict the distribution of these movements under tightly controlled experimental conditions. Behavior, however, is precisely an act of resistance against a changing environment (Marken, 2009) and there is thus a “tug-of-war” between the experimenter who wants to control the environment of the animal and the animal who wants to have control over that same environment. For this reason, researchers often find themselves heavily constraining the opportunities for an animal to win over that control, to reduce the variability in animal movement. Head-fixation or even anesthesia are used both to make it easier to measure internal physiological state and to simplify the analysis of the behavior. However, such extreme conditions can fundamentally change the nature of the relationship between the animal and its environment, with corresponding changes in the dynamics of neurophysiological activity and a non-negligible impact in the interpretation of experimental results.

In this article, we consider two alternatives to the classical head-fixation or anesthetized paradigms: virtual fixation and voluntary fixation. We discuss how these approaches can resolve fundamental issues in the design and analysis of behavioral experiments and introduce an emerging set of modifiable open-source tools aiming to make them increasingly more accessible to behavioral researchers. We include practical examples and tutorials using the Bonsai visual programming language to help researchers immediately start applying these methods to analyze and study brain circuits and behavior (see also Supplementary Material for basic tutorials).

This piece is part of the research topic “New Insights into Behavioral Pharmacology,” organized as an extended forum for the workshop “From networks to behavior and back,” a satellite event of the European Behavioral Pharmacology Society (EBPS) Biennial Meeting (August 2019, Braga, Portugal).

### Virtual Fixation

**Figure 1:**
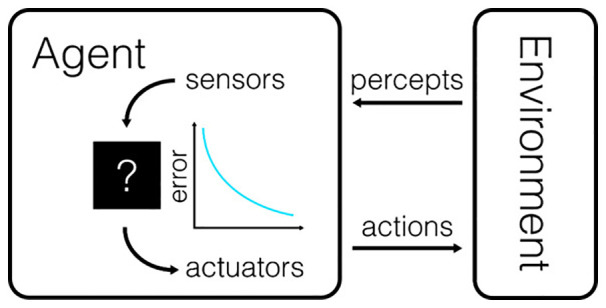
The canonical model for the interaction between a behavioral subject and its environment. The agent receives information about the state of the environment through a variety of sensors and can control the state of the environment through the use of actuators. Actions are chosen to minimize the difference between the perceived state of the world and the intended state of the world. The amount by which this difference is reduced is also sometimes referred to as the utility of an action.

One of the gold standards for tests of causality is reproducibility: any putative relationship between a causal variable and its effect should be reliably observed. To pick up on such statistical regularities, researchers try to establish comparable conditions under which the relationship can be recorded many times. In behavioral studies, it is common to consider the mapping between “perception” and “action.” Perception is the information about the state of the environment that is accessible to the animal at any given time, and action is the set of movements performed by the animal to change its relationship with the environment (Figure 1).

To reduce the variability of this complex interaction, researchers often go to great lengths to constrain the variability in perception, in the hopes of reducing irrelevant variability in possible actions. Ironically, to do this effectively requires the action of the animal to be constrained in the first place, often by head-fixation, since even small changes to the position or orientation of the head relative to the environment will dramatically change the amount and type of stimulation reaching the sensors.

An alternative to head-fixation is the freely moving paradigm. In this situation, the animal is free to move in the environment, thus reinstating control over its perception. This situation is often considered by neurophysiology researchers to be a “harder” setting for behavioral neurophysiology, as precise control over the input stimulus is lost.

Surprisingly, however, the neurophysiology and mechanisms of specific brain systems, such as hippocampal navigation, only really started to emerge by allowing animals to be observed under freely moving conditions (O’Keefe and Dostrovsky, 1971; Kandel, 2014). The discovery and analysis of place cell activity rely on the ability to monitor neuronal firing during freely moving navigation. Rather than constraining the input/output mapping between stimulus and action, the researcher records the spontaneous behavior of the animal in detail together with the physiological data and then correlates neural activity across comparable conditions during the experiment, such as the animal location in space. We call this paradigm “virtual fixation.”

In virtual fixation, the goal is to identify reproducible conditions by precise continuous measurement of animal behavior over time. By identifying moments where the conditions of interest can be reliably compared, behavior becomes amenable to statistical analysis despite occurring spontaneously. Perceptual states can thus be fixed for analysis without artificially fixing the subject or the stimulus.

Of course, this approach is highly dependent on the accuracy of our behavioral measurements, and on the perceptual states we are interested in. Tracking the center of mass position of a single animal in the open field is enough to reconstruct reliable place cell activity. However, it is not enough if we are interested in measuring the distance between the nose and an object, the postural angles of the limbs during walking, or in knowing how many photons are hitting the retina at a given time point.

Fortunately, emerging data analysis techniques are expanding the scope of possible conditions which are amenable to virtual fixation. The use of machine learning technology has entered full-force into behavioral labs worldwide through the introduction of tools such as DeepLabCut (Mathis et al., 2018), which can lower the cost for tracking any user-labeled feature in video datasets. If the human eye can identify a feature of interest in a video, there is now a good chance we can automatically derive a tracker to reliably extract that feature for analysis. Markerless limb and body part video tracking used to be an approach limited to highly technical laboratories which is now much more accessible due to the open-source nature of these tools. Furthermore, first-order features can often be combined to yield other measures of interest. For example, tracking head position can be used to infer what portion of the visual field is accessible to the animal at each moment, thus allowing the researcher to precisely determine which visual information is accessible to the animal, despite freely moving conditions (Walter and Couzin, 2020).

### Virtual Fixation in Practice

Until recently, the development and application of machine learning tools have required moderate programming experience (often in Python), or otherwise relying on standardized video analysis toolkits with a limited set of functionalities. However, the broad applicability of these techniques to diverse datasets has triggered widespread interest even in communities of researchers with no explicit technical training in computer science or computer vision.

Given the diversity of researchers’ interests and the difficulties outlined above in defining what is behavior from a measurement perspective, such tools must ideally combine flexibility with ease of access for non-experts. For this tutorial, we will rely on the visual programming language Bonsai^1^ (Lopes et al., 2015) to illustrate some basic principles of freely moving behavior measurement, analysis, and virtual fixation.

Although many approaches can be used to measure ongoing animal activity, we will start with video analysis as it remains the tool of choice for non-invasive, flexible, and unbiased investigation of behavior (in contrast to “lever presses” and “nose pokes,” the video does not entail too many assumptions on what behavior is before making a measurement). It also does not require complicated hardware setups, as cameras can now be acquired very cheaply and can be placed virtually anywhere, provided that an adequate view of the animal can be obtained. Illumination and occlusion certainly pose a fair share of problems, but existing extensive collections of resources on photography and videography can help researchers to understand and resolve the majority of these issues.

**Figure 2:**
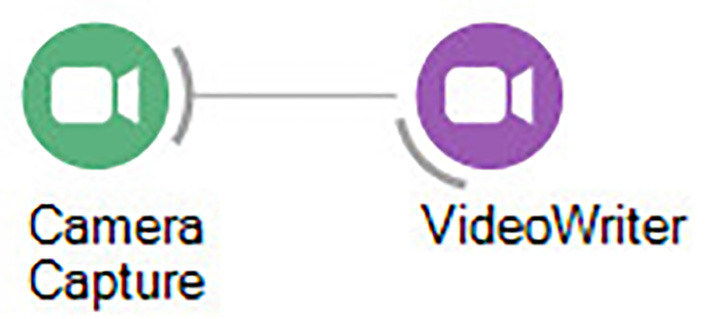
Bonsai workflow for recording video from a single camera.

Once the setup is in place, and a compatible camera is connected to the computer, video can be acquired in Bonsai with a simple workflow (Figure 2).

**Figure 3:**
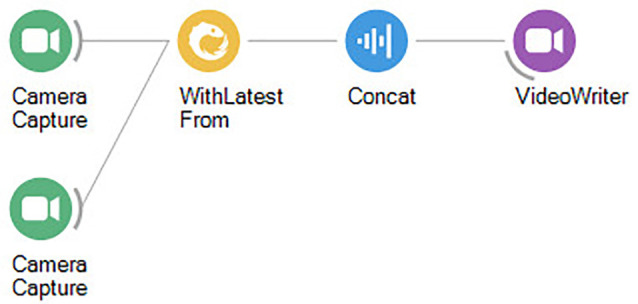
Bonsai workflow for correlating video from two cameras simultaneously.

It can often be helpful to record videos from multiple perspectives to gain more information about the contingencies surrounding the animal in the freely moving condition. In this case, care should be given to ensure that it will be possible to correlate information from multiple perspectives frame-by-frame. This is possible in single-camera setups by using mirrors to direct light from different angles to the same sensor. In multi-camera setups, we need to correlate the image acquisition in time to ensure that information from one camera can be matched to information from the other cameras. Camera models supporting digital triggers can be used to precisely synchronize the exposure of each frame to an external pacemaker. Otherwise, we can correlate multi-camera acquisition in software by taking the latest exposure from the extra cameras, every time a new frame is collected (Figure 3).

Following video recording, data can be processed offline (see Supplementary Material for basic tutorials), but all examples shown here can also be used verbatim online to make real-time decisions on the conditions presented to the animal during closed-loop experiments (see “Voluntary Fixation” section).

**Figure 4:**
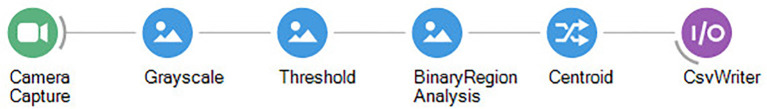
Bonsai workflow for tracking the position (centroid) of a single animal in a well-lit arena, assuming a simple contrast threshold (black on white background).

Virtual fixation requires the reliable extraction of features from the video which we can use to establish comparable conditions for data analysis. By far the most commonly used feature is the spatial position of the animal (usually the center of mass), referenced to a fixed set-up (often a box or arena). The reason for such popularity can be justified by how much information can be derived from this simple metric relative to how easily it can be retrieved from the video (Figure 4).

Manipulating the resulting time-series of 2D positions can be used to gain insight into the recorded behavior. Binning the data spatially can generate occupancy maps, thus indicating where the animal spends most of its time; the numerical difference over time will give an approximation for speed or quantity of motion, thus indicating when the animal is active or quiet; and defining spatial entry or exit conditions allows identifying moments where the same path was taken (e.g., in a maze). If physiological data is available and synchronized with the video, it becomes possible to correlate animal position in the arena with physiological signal patterns (e.g., a spike from a specific cell); or the converse, what is the pattern of brain activity when the animal decides to enter a specific area within the arena.

**Figure 5:**
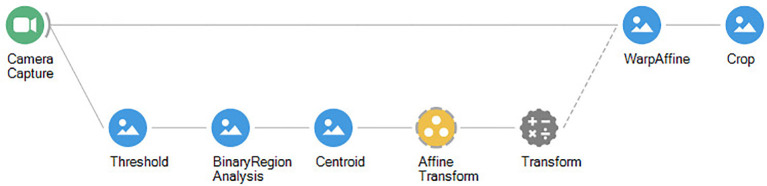
Bonsai workflow for dynamic cropping of a region of interest around the center of the animal.

**Figure 6:**
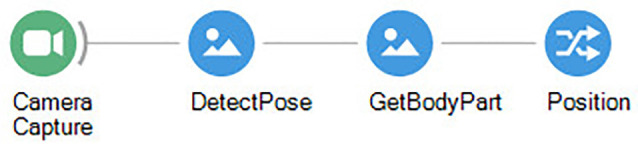
Bonsai workflow for tracking a specific body part using the Bonsai-DLC package.

By feeding back the result of tracking it is possible to dynamically crop a region of interest around the subject to obtain an ego-centric video where the animal is always in the center (Figure 5). From there we can analyze the video consistently for proximal cues surrounding the animal at any moment, at any point in the arena. For example, a common application of this strategy is to extract a sequence of frames used for training feature detectors by machine learning libraries such as DeepLabCut (Mathis et al., 2018). This way we can refine our initial tracking to include specific body parts such as the nose, ears, or paws (Figure 6). Furthermore, cropping the video will reduce the dimensionality of the data, therefore significantly reducing training time and speeding-up inference performance for online analysis.

Expanding the basic center of mass tracking to specific body parts, using DeepLabCut or other simple image processing techniques, expands the possibilities for virtual fixation even further, allowing fixing specifically the video around the nose or head of the animal, and then calculating distances between those body parts and other points of interest (or other animals; Kane et al., 2020).

Ultimately, even failure modes in tracking can be interesting. If the tracking is well-calibrated or trained for a specific behavior pattern, then unexpected failures can work as outlier detectors and may well reveal moments of interest in the video (where the animal displays a novel behavior pattern or otherwise deviates from the usual path). In these situations, having an exploratory data analysis workflow with the ability to go from temporal information about a tracking failure back to the video can be extremely useful to assist with interpretation.

### Voluntary Fixation

Behavior is often considered to be intrinsically variable, but the whole history of motor control tells us that the story is slightly more complicated than that (Lopes and Kampff, 2015). Behavior and motor control in most animals are incredibly precise, depending instead on the constraints of the body, the task, and most importantly, what the animal is trying to achieve. Interestingly, what tends to happen during training is that animals will shape their degrees of freedom around the exact constraints required to succeed at the task, leaving everything else variable. It is common for example in high temporal precision lever pressing tasks to see animals develop extreme stereotypical patterns which unfold through extremely reliable sequences, and yet will be highly idiosyncratic across individuals (Kawai et al., 2015).

We can observe this proposition by contrasting variability in movements (high), to variability in controlling desired state (not high). Animals will eat, drink and sleep when necessary in extremely reliable patterns, although the means to achieve those ends might be highly variable. If constraints are introduced that need to be overcome to achieve their goals, animals will reliably overcome them, even if the means to do so might surprise and frustrate the researcher. Means are variable, ends are less so, and indeed a large part of behavior is resisting external perturbations, no matter the cost, to achieve goals reliably.

**Figure 7:**
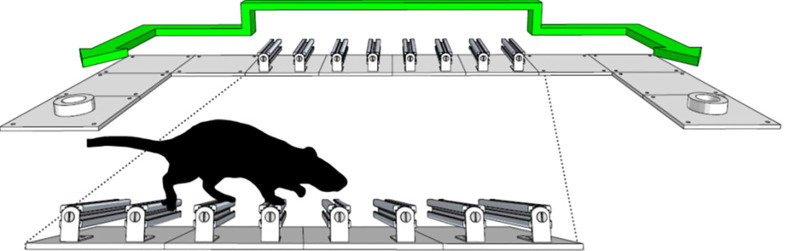
Schematic of the shuttling box apparatus. Animals will forage for liquid reward in this environment, either sucrose or water if using water restriction, and will readily shuttle between the two ports (adapted from Lopes et al., 2016).

It is thus not surprising to find researchers relying on naturally expressed drives and behaviors to challenge the animal to engage with the researcher’s experimental apparatus. Water restriction or aversive stimuli remain common motivators of action in behavior studies such as the shuttling box (Figure 7). Another, arguably more humane, approach is to exploit the flexibility of the animal to adopt different goals, and indeed to be trained for different tasks using operant conditioning, where the animal is trained using secondary reinforcers to respond to specific stimuli in ways which are of interest to the researcher.

Both operant conditioning and intrinsic drives have been widely exploited in experimental psychology and experimental neuroscience as means of creating reproducible conditions. For our purposes, we group the collection of both approaches under the term “voluntary fixation.” The main goal of voluntary fixation is getting animals to constrain their behavior without the need for pharmacological or physical restriction. Indeed, in some extreme cases, this might be the only way to even approach *in-vivo* fixation studies themselves. For example, rats are notoriously difficult to restrain in awake head-fixed preparations, as they will leverage their incredible arm strength and often damage their own skull in an attempt to escape. But recent self-fixation studies have shown that rats will happily self-fix in awake imaging apparatuses in exchange for a reward, as long as it remains their own self-paced decision to do so (Scott et al., 2013).

As with virtual fixation, voluntary fixation places the burden on the researcher to design experimental apparatuses which will work with the animal, either accommodating their natural drives or using automation to deploy operant conditioning protocols where the animal can learn to constrain its behavior in exchange for a reward. In both cases, the technological investment can be too much to bear. Designing automated boxes for behavioral studies requires a coordinated combination of mechanical, electronic, and software design skills which can challenge even the best engineers, and for that reason used to be out of reach for the majority of experimental neurobiology and behavioral pharmacology researchers.

### Voluntary Fixation in Practice

Following the advent of accessible hobby electronics platforms such as the Arduino^2^, and the opening up of Ph.D. programs to multi-disciplinary candidates, neuroscience has now grown a healthy community of hackers and rapid-prototyping aficionados. Those early researchers who were sympathetic to the open-source and open-science movements ended up adopting those engineering practices while developing their work, resulting in open platforms and tools developed and shared broadly across the neuroscience community. These open devices can now be quickly assembled for monitoring animal actions such as licking and lever pressing or controlling the environment using motors, lights, and sounds (Freeman, 2015; White et al., 2019).

The challenge now remains on how to combine the operation of all these devices in a way that is easy to understand and customize for individual experiments. Ideally, we further want to combine this control with other tools for rich monitoring of behavior such as the techniques for virtual fixation based on the video discussed above. Most interfaces require bespoke programming skills to achieve the required control over the precise, moment to moment, the sequence of events in our experiment, and support closed-loop interactions, so we will again use Bonsai to design some simple, yet functional, illustrations of automated environments which can support voluntary fixation paradigms.

**Figure 8:**
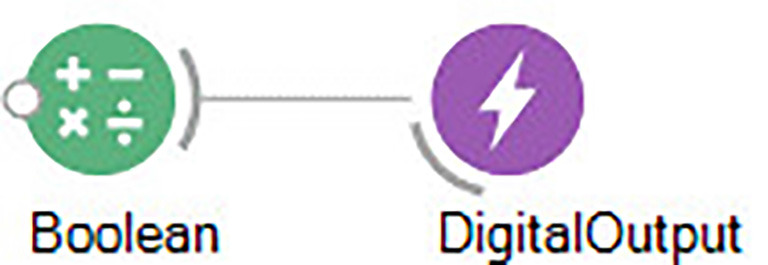
Bonsai workflow for controlling a single Arduino digital output.

We will start by changing different aspects of the environment using an Arduino microcontroller, which provides different digital output ports which can be controlled directly in Bonsai with a simple workflow (Figure 8).

The state of each port can be modified simply by changing the state of Boolean inputs which can be either *True* or *False*. These will correspondingly change the voltage at the terminals of the Arduino between +5 V and 0 V, which can be used to turn lights or lasers on or off, trigger reward, or open valves.

**Figure 9:**
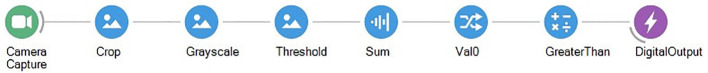
Bonsai workflow for triggering a digital output based on a region of interest.

To support voluntary fixation, we need such changes to be triggered automatically so the animal can control the environment using its behavior. This means we need to be able to compute a change in logical level while the animal is moving freely in the environment. For example, to emulate the above foraging patches we can specify regions of interest in the arena which will activate a port whenever an animal enters the patch (Figure 9).

**Figure 10:**
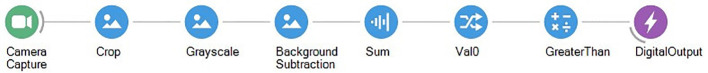
Bonsai workflow for triggering a digital output based on movement.

Changing the nature of the electronic devices wired to the Arduino allows a broad range of automated responses to be designed, from triggering lights and sounds to electrical or optogenetic stimulation. The online calculation can also easily be changed to operate on other features of animal behavior, such as the amount of motion (Figure 10), which will allow for output stimuli to become contingent on animal freezing or fast movements.

Such dynamic control procedures operated using low latency feedback allow the design of environments with interactive properties which can be directly operated by the animal in real-time. Crucially, the experimenter is now in control of the interaction and can modify the response characteristics of the closed-loop system to investigate animal behavior, for instance by delaying, suppressing, or amplifying the feedback response parametrically. Using Bonsai, virtual fixation techniques can themselves be used in the design of such closed-loop systems, for example by using the increasing computational capabilities of GPUs for real-time pose estimation (Kane et al., 2020).

**Figure 11:**
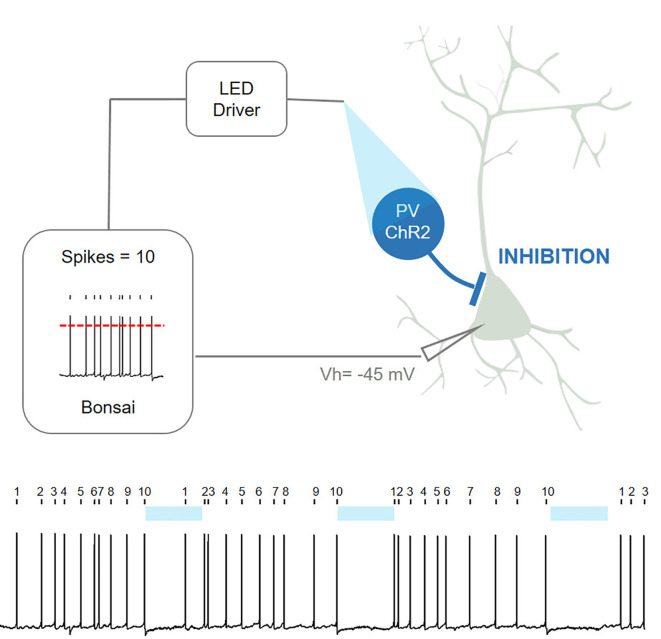
Closed-loop patch-clamp experiment with Bonsai (contributed by Gonçalo Lopes, André Marques-Smith, Luis Jacinto, and Patricia Monteiro [unpublished]).

Closed-loop systems are also not restricted simply to behavior and can be successfully used even for purely physiological investigations in living nervous systems. In the example shown below, Bonsai was used for a patch-clamp closed-loop experiment (Figure 11). *Ex-vivo* brain slices were prepared from transgenic mice expressing channelrhodopsin (ChR2) in cortical inhibitory interneurons [parvalbumin-positive interneurons (PV)]. Neighboring cortical pyramidal neurons (without ChR2) were patched to record spontaneous firing activity and Bonsai was used to count the number of action potentials fired by the pyramidal neuron in real-time. Upon every 10th action potential, Bonsai triggered a 488 nm fiber-coupled LED, leading to optogenetic stimulation of PV interneurons and inhibition of pyramidal neurons. This design can impose a new self-paced firing pattern where a period of ten action potentials is followed by a period of silence (5 s optogenetic induced silencing) using continuous real-time closed-loop feedback.

**Figure 12:**
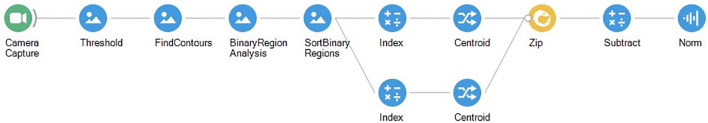
Bonsai workflow for measuring the distance between the two largest objects.

Finally, voluntary fixation paradigms can be extended not just to the study of the relationships between a single animal and its environment, but also to interactions between multiple animals. This is often perceived as much harder given the difficulty in identifying individual animals without complex computer vision algorithms. However, the use of simple features relying on invariant geometric properties can yield behaviorally meaningful and surprisingly robust metrics. For example, the following workflow will compute the distance between two animals in a single arena (Figure 12).

Since distance is a commutative quantity, we completely avoid the need to uniquely identify each animal, and thus easily achieve fast, real-time performance. By coupling this quantity to a digital output port in the Arduino in the same way as the above examples, we would now be able to trigger stimulation contingent on the distance to a conspecific.

## Conclusion and Future Perspectives

In numerous tasks involving operant conditioning and intrinsic drives have been widely used in experimental psychology and neuroscience to reproducibly study animal behavior. Back in 1959, David Premack proposed that reinforcers should be seen not as stimuli but rather as opportunities to engage in a behavior (Premack, 1959). In his famous drinking vs. running experiment, he showed that thirsty rats prefer to drink rather than to run in the wheel, but when rats are not thirsty, they prefer to run rather than to drink. In other words, the activity should be regarded as the reinforcer, not the stimulus of water (Premack and Anglin, 1973). Of relevance to this discussion, Premack suggested that animals should be allowed to engage freely in activities. Accordingly, as behavioral neuroscientists, we should consider factors that may determine when, and how vigorously, responses will be freely performed and how exploitation of new tools and new behavioral paradigms might grant us experimental control over those responses.

New technologies and open-source tools for neuroscience are rapidly pushing the boundaries of what we can study, and how we study, the brains of awake behaving animals (Freeman, 2015; White et al., 2019). Opposite to proprietary tools that present limited collaborative development and restricted experimental designs, open-source tools allow customization and flexibility. Since its publication in 2015 (Lopes et al., 2015), Bonsai has been widely used by many labs worldwide not only for tracking animal behavior in different species of rodents, cephalopods, fish, and insects (Dreosti et al., 2015; Walker et al., 2015; Douglass et al., 2017) but also to control and acquire data from multiple streams. Being open-source software, Bonsai is free to use and not proprietary to any one company, thus many users have adopted it to create their specific packages for EEG (Lopes, 2018), Miniscopes (Aharoni and Hoogland, 2019; Guo et al., 2020), Fiber photometry (Carvalho and Lopes, 2019), Open Ephys (Neto et al., 2016) and real-time video analysis with DeepLabCut (Kane et al., 2020). It also encourages good practices for experimental reproducibility by including a built-in package manager and support for portable deployment across rigs. This means that experimental workflow environments can be shared across labs while ensuring the behavior control software is reproducible. This has been leveraged with great success on large international collaborative projects such as the International Brain Laboratory (The International Brain Laboratory et al., 2020).

Leveraging on this exciting open-source hardware/software ecosystem, it is now possible to study animals’ naturalistic behaviors while maintaining control over many other variables, and potentially also integrating it with large-scale housing environments (Castelhano-Carlos et al., 2017). In other words, instead of letting existing behavioral paradigms drive the research question, scientists can now design and implement custom behavioral neuroscience experiments with unprecedented control and intellectual freedom.

There are, however, challenges ahead. Because of their flexible nature and large degrees of customization freedom, open-source tools will always require some troubleshooting and experimental validation. Although user community help and development are more and more available, technical support for specific user problems might not be readily available. To overcome this challenge, we need to foster stronger communities and platforms for dissemination, sharing, and training in these open technologies. Only by understanding how such tools work can researchers fully leverage their advantage and realize a healthy open-source mindset for neuroscience. Towards this goal, it is also fundamental that funding agencies start to support and incentivize these development and dissemination efforts, which currently still rely mostly on the passion and determination of lone researchers to share the results of their work, often at great cost to their professional careers as researchers. To protect the future of open-source tools, the academic science community needs to recognize and value such contributions themselves.

Despite being in its infancy and despite all the above challenges, open-source tools have already demonstrated the benefits of shared neuroscience and currently play a significant role in the field of behavioral neuroscience. Their future is bright and adopting a collaborative mindset for the behavioral neuroscience field will prove itself crucial to driving our understanding of the brain. Ultimately, though, studying the neural basis of behaviors still depends on the ability to design the key experiment. It is up to researchers to ask the right questions.

## Data Availability Statement

The original contributions presented in the study are included in the article/Supplementary Material, further inquiries can be directed to the corresponding author/s.

## Author Contributions

GL and PM conceptualized and wrote the entire manuscript. All authors contributed to the article and approved the submitted version.

## Conflict of Interest

GL is director at NeuroGEARS Limited. The remaining author declares that the research was conducted in the absence of any commercial or financial relationships that could be construed as a potential conflict of interest.

## Footnotes

^1^All schematics included in the tutorial are fully functional workflows for the Bonsai programming language which can be executed in the development environment which is freely available online at https://bonsai-rx.org.

^2^Arduino is an open-source electronic prototyping platform described in more detail at https://www.arduino.cc/.
