# Supplementary material for: New Open-Source Tools: Using Bonsai for Behavioral Tracking and Closed-Loop Experiments
Source: Front Behav Neurosci. 2021 Mar 31;15:647640. doi: 10.3389/fnbeh.2021.647640 (PMC8044343; doi:10.3389/fnbeh.2021.647640)
Supplement: Supplementary file 1 [file Data_Sheet_1.PDF]

Bonsai Basic Tutorial #1: Acquiring and saving a video

Bonsai can be used to acquire and record data from many different devices. The most common input device is perhaps a video camera used for behavioral observation. So, how to perform video acquisition with Bonsai? First, you need to have a camera plugged into an USB port on your computer. Download Bonsai from http://bonsai-rx.org and install 'Bonsai - Starter Pack' from the package manager. Once you open Bonsai and start a New Project, you will see three distinct panels from left to right: 'Toolbox panel', 'Workflow panel' and 'Properties panel' (Fig.S1).
On 'Toolbox panel' search for the 'VideoCaptureDevice' source and insert it as a node onto the 'Workflow panel' by double-clicking on it or by dragging it onto the 'Workflow panel'. Now if you hit the 'Start' button and double-click on the 'VideoCaptureDevice' node, you will see what that node is doing (in this case, displaying your video image). To save the video, stop the Workflow and insert a 'VideoWriter' sink node. Connect the previous node ('VideoCaptureDevice') with the new node ('VideoWriter') by dragging it on top of each other (a straight horizontal line will appear connecting both nodes). Then click on the 'VideoWriter' node, go to 'Properties panel', and click on 'FileName' to choose a folder to save your video. The video should be saved in avi format by typing a FileName such as 'MyVideo.avi' (.avi file extension). Run the Workflow (Fig.S1) and check that it generates a valid video file on your selected folder.

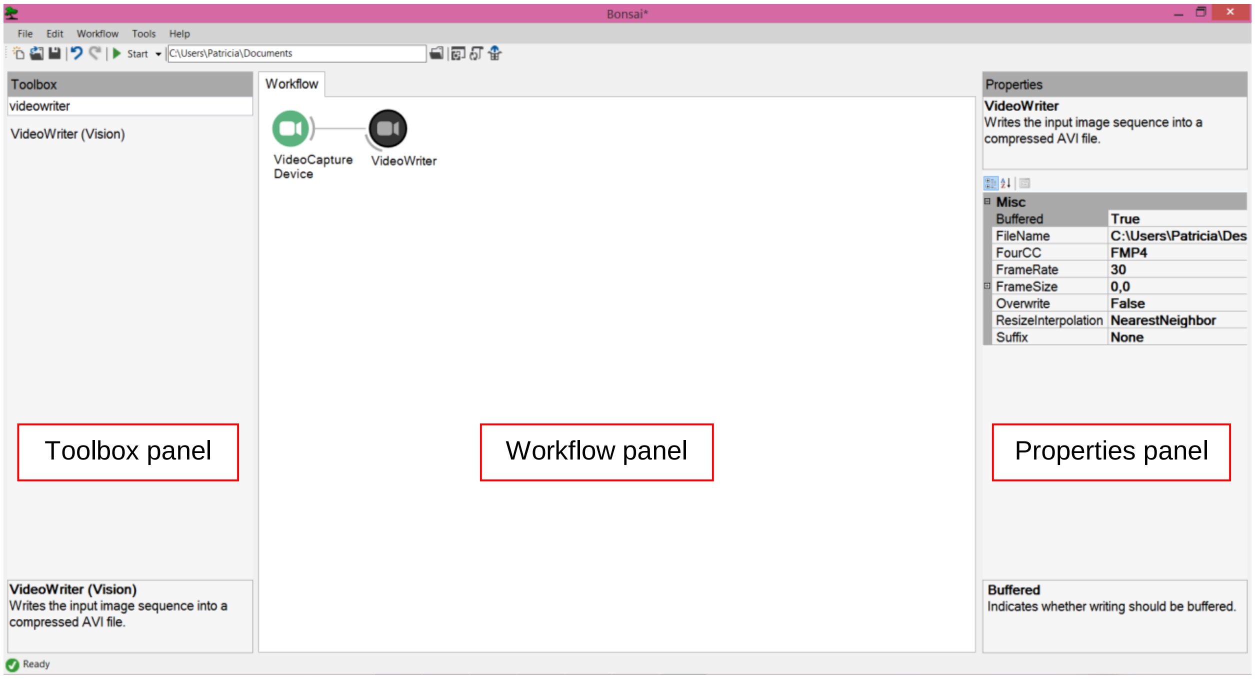

Figure S1 - Acquiring and saving a video with Bonsai GUI

Bonsai Basic Tutorial #2: Animal tracking

So let's assume you have now recorded a video of an animal freely moving inside an arena. Or perhaps you have a video from an animal that you recorded last year. How to perform animal tracking with Bonsai?

Start a New Project on Bonsai and insert a 'FileCapture' source node from the 'Toolbox panel'. Next, on 'Properties panel', click on 'FileName' to indicate the video directory (the location where the behavioral video is saved on your computer). You can also directly double-click on the node. Remember that the video file should be avi format (.avi file extension). Now if you hit the 'Start' button and double-click on top of the 'FileCapture' node, you will see what that node is doing (in this case, displaying your previous behavioral video) (Fig.S2.1).

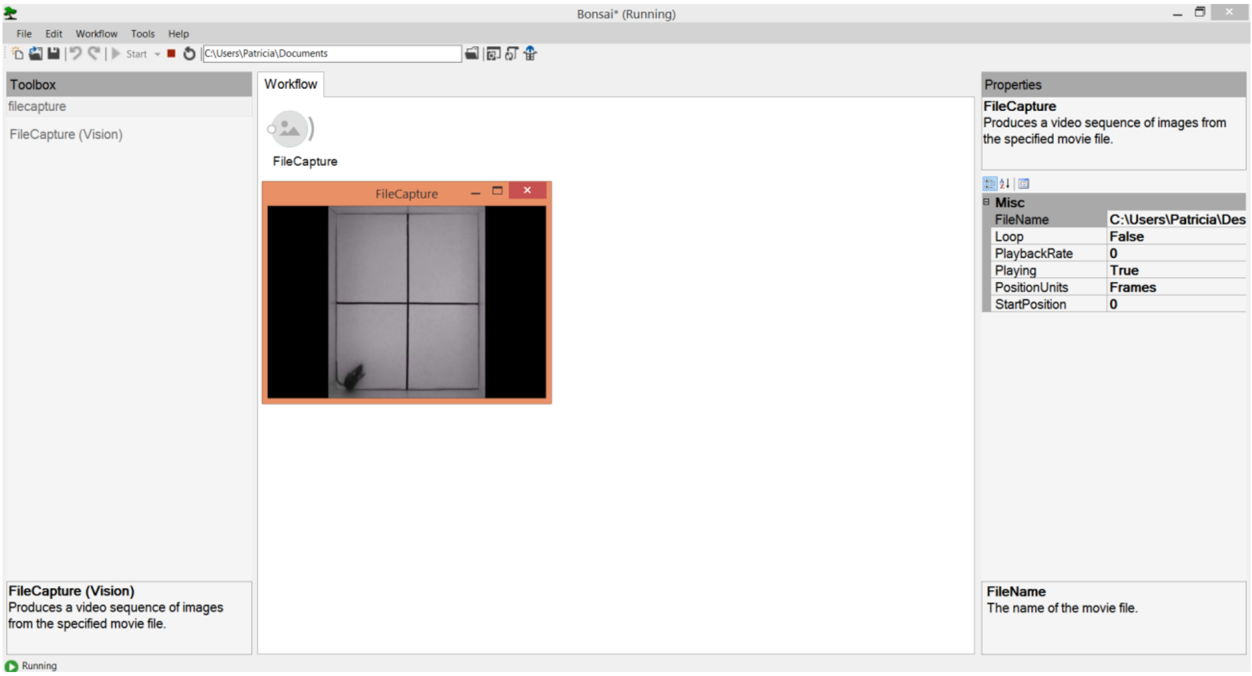

Figure S2.1 - Loading a video with Bonsai GUI

Next, insert a 'ConvertColor' transform on your 'Workflow panel' and then an 'HsvThreshold' transform. Hit the 'Start' button and double-click on all the nodes to visualize what each node is doing. To isolate your animal, click on the 'HsvThreshold' node on your 'Workflow panel' and then configure its Lower and Upper values on the right 'Properties panel' (Fig.S2.2).

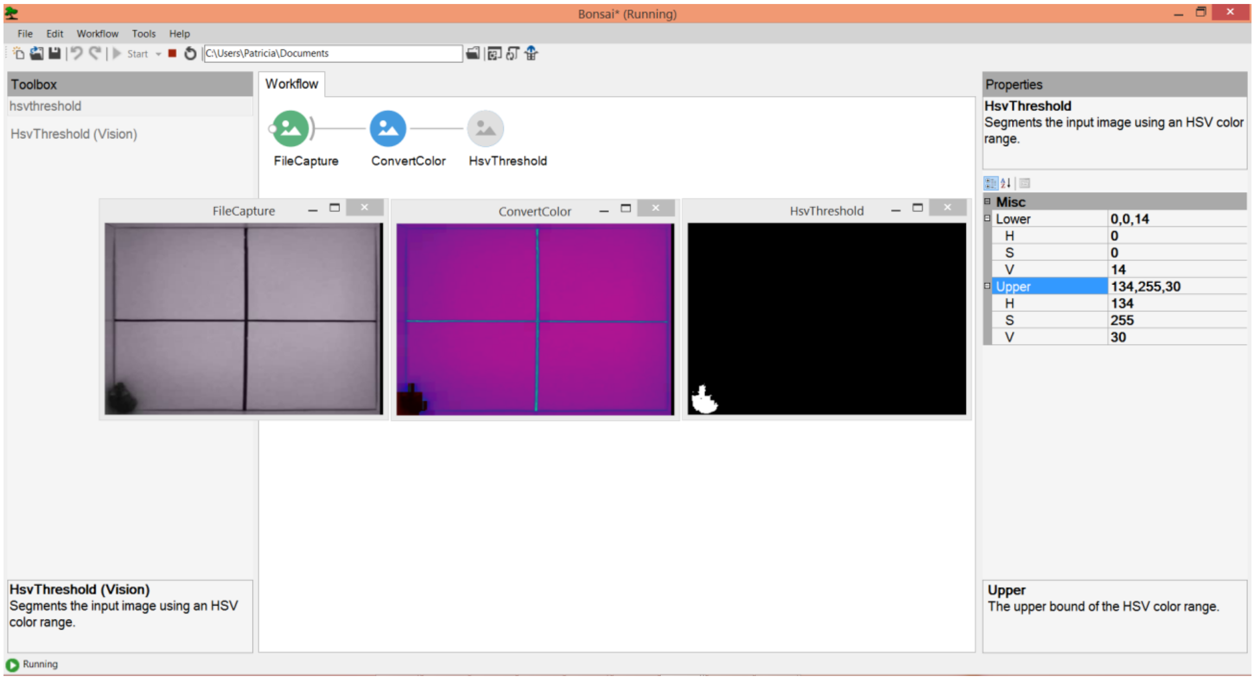

Figure S2.2 - Thresholding a video for animal tracking with Bonsai GUI

Once you find appropriate threshold levels, stop the workflow and insert a 'FindContours' transform on your 'Workflow panel'. This operator traces the contours of all the objects in a black-and-white image. An object is defined as a region of connected white pixels. Then, insert a 'BinaryRegionAnalysis' transform. This node calculates the area, center of mass, and orientation for all the detected contours. Then, to extract the largest detected object in the image (your moving animal) insert a 'LargestBinaryRegion' transform, right click on it and select the option 'Output' > 'Centroid'. Lastly, record the position of the centroid by inserting a 'CsvWriter' sink at the end of your Workflow. Don't forget to give it a filename by clicking on the 'CsvWriter' node and adding a filename such as 'MyResults.cvs' in the 'Properties panel'. All set. Now when you hit 'Start' the centroid position of your animal will be tracked and saved (Fig.S2.3).

Pro-tip 1: when you hit 'Start', drag the 'centroid' node on top of your video ('FileCapture' window) to visualize the centroid in real-time. You can also now click on the new centroid in the 'FileCapture' to see the movement track left by your animal while exploring the arena.

Pro-tip 2: you can replace the 'FileCapture' node by a 'CameraCapture' source to track animals in real-time during video acquisition, instead of tracking animals from a pre-recorded video.

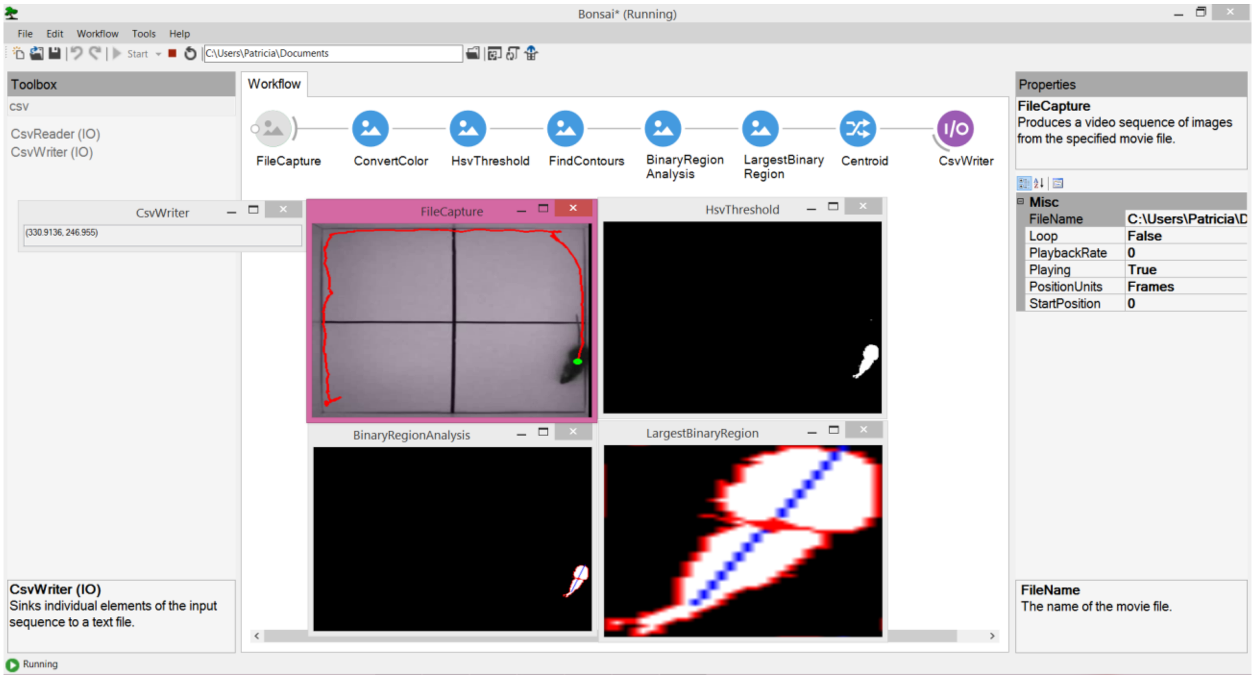

Figure S2.3 – Simple animal tracking (black on white background) with Bonsai GUI

Bonsai Basic Tutorial #3 - Animal tracking in complex backgrounds

Not all behavioral tasks consist of dark rodents placed inside a white background arena. Sometimes fur colors are diffcult to differentiate from background colors and from other objects. For those cases, researchers can use background subtraction followed by motion tracking with Bonsai. The only requirement is that your video must start with the background before inserting the animal. Bonsai will take a snapshot of that first background image and subtract it to the remaining video.

Create a New Project on Bonsai and insert a 'FileCapture' node (don't forget to indicate the video FileName) and a 'Grayscale' node. Now insert a 'Skip' operator node and set its 'Count' property to 1 on the right 'Properties panel'. In a new branch, insert a 'Take' operator (set its Count property to 1) and connect this node to the ''Grayscale' node. Combine the images from both branches using the 'CombineLatest' combinator node. Insert the 'AbsoluteDifference' transform node after 'CombineLatest'. Insert a 'Threshold (Vision)' transform node. Hit 'Start' and visualize 'FileCapture', 'AbsoluteDifference' and 'Threshold' nodes by clicking on it. Adjust the 'ThresholdValue' property. Now, just add the remaining Worflow previously described in the animal tracking section: 'FindContours', 'BinaryRegionAnalysis', 'LargestBinaryRegion'>Centroid, 'CsvWriter'>Filename (Fig.S3).

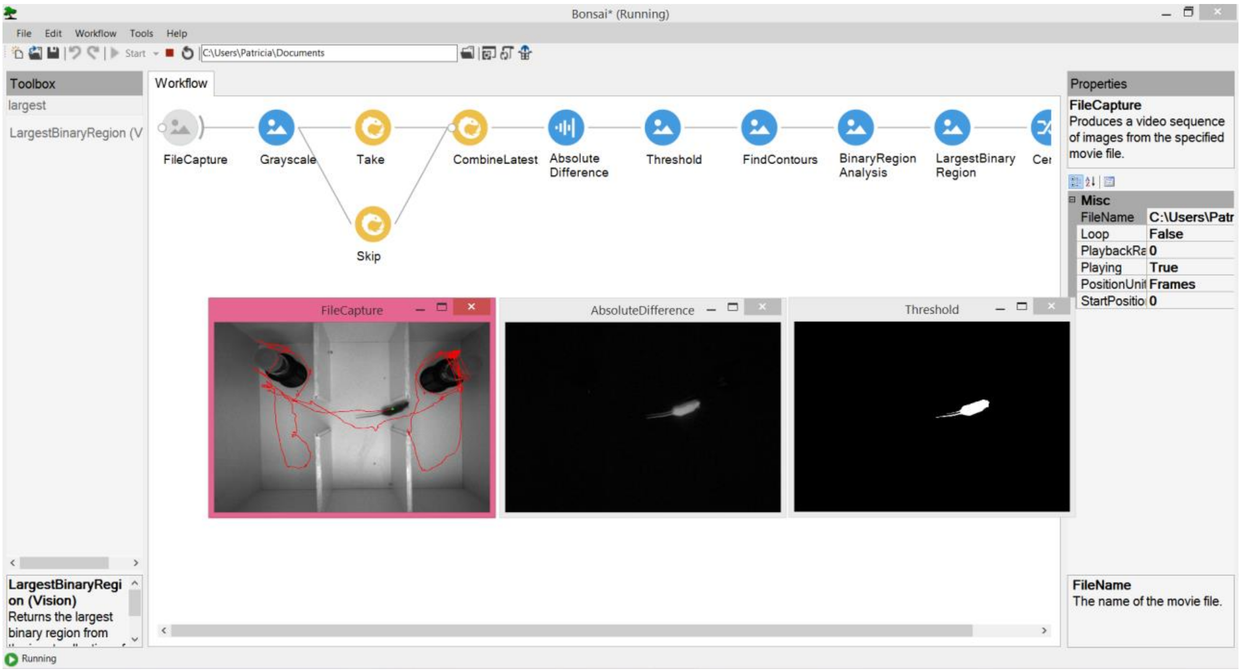

Figure S3 – Animal tracking in complex background with Bonsai GUI
